# Faces and phases of epistemic curiosity in science learning: A longitudinal study

**DOI:** 10.1111/bjdp.70041

**Published:** 2026-03-22

**Authors:** Susanne Koerber, Christopher Osterhaus

**Affiliations:** ^1^ Freiburg University of Education Germany; ^2^ University of Vechta Germany

**Keywords:** epistemic curiosity, longitudinal study, primary school, science (physics) knowledge, scientific reasoning

## Abstract

Curiosity is central to children's development, particularly in science learning. This longitudinal study examines how epistemic curiosity, overall and its subtypes I‐type and D‐type, predicts scientific reasoning and science (physics) knowledge at the start (Grade 1) and end (Grades 3–4) of primary school, beyond prior knowledge and cognitive abilities. Parents of 122 children (mean age 6.12 years) completed an 11‐item curiosity questionnaire. Scientific reasoning and science knowledge were assessed with standardized inventories and a 25‐item physics test. Overall curiosity predicted early reasoning, whereas I‐type curiosity predicted later science knowledge. These findings highlight that curiosity has distinct faces and phases: I‐type and D‐type operate differently at different developmental time points. Fostering both types supports multiple aspects of science competence.

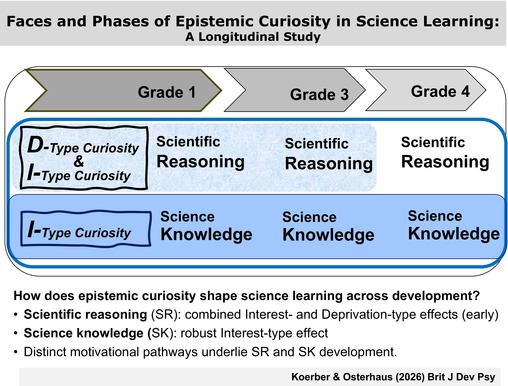

## Graphical abstract

Different faces of epistemic curiosity shape science learning across development. While early scientific reasoning depends on the joint action of interest‐type and deprivation‐type curiosity, science knowledge is robustly driven by interest‐type curiosity, revealing a developmental reweighting of motivational mechanisms.

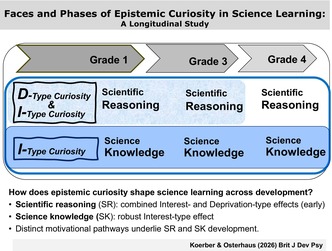

**Statement of Contribution**

**What is already known on this subject?**

- Scientific reasoning is a core cognitive competence developing across early to middle childhood.
- Curiosity supports cognitive and motivational development and includes I‐type and D‐type forms.
- Longitudinal research linking epistemic curiosity to science competencies remains limited.

**What the present study adds?**

- Epistemic curiosity has *faces* (I‐type, D‐type) and *phases* (different roles across development).
- Scientific reasoning is predicted early by I‐ & D‐type; science knowledge across school by I‐type.
- Curiosity predicts science learning beyond prior knowledge and intelligence.

Scientific reasoning is a core component of children's cognitive development (Kuhn, 2011; Zimmerman, 2007) and a central pillar of scientific literacy. It involves generating hypotheses, evaluating evidence and drawing inferences (Sodian, 2018), and it supports the acquisition of science knowledge across the school years (Koerber & Osterhaus, 2019). Even in early childhood, basic experimentation and evidence evaluation can be observed (Piekny & Maehler, 2013). Longitudinal work shows that these early competencies are relatively stable and predict later outcomes in science and related domains (Koerber & Osterhaus, 2019; Osterhaus & Koerber, 2025).

Most research on the development of scientific reasoning has focused on cognitive predictors, including intelligence, language skills, theory of mind and executive functions (e.g., van der Graaf et al., 2018). In contrast, motivational and affective factors have received comparatively less attention, although they are increasingly recognized as crucial for sustained engagement and learning in science (Bjerknes et al., 2023; Fischer et al., 2018; Jirout et al., 2024; van Vo & Csapó, 2023). Among these factors, epistemic curiosity has emerged as a promising candidate for explaining why some children invest more effort in scientific activities and accumulate more science knowledge than others (Hartung et al., 2022; van Schijndel & Jansen, 2025).

Epistemic curiosity refers to the intrinsic desire to acquire new information and to reduce uncertainty, and it is considered a key driver of exploration, questioning and self‐initiated learning (Litman, 2005). As an epistemic emotion, curiosity signals perceived knowledge gaps and motivates cognitive engagement to resolve them (Litman, 2008; Loewenstein, 1994). This mechanism aligns with models of self‐regulated learning and metacognitive control, which emphasize that motivation and monitoring jointly guide strategic information seeking and problem solving (Pintrich, 2000; Veenman, 2013). Because scientific reasoning relies on similar processes and requires sustained attention to ambiguity, evidence evaluation and flexible revision of ideas (Kuhn, 2011; Sodian, 2018), epistemic curiosity may provide the motivational foundation for engaging in these demanding cognitive processes.

A crucial distinction within epistemic curiosity is between interest‐type (I‐type) curiosity, driven by the intrinsic enjoyment of learning, and deprivation‐type (D‐type) curiosity, driven by an aversive feeling of not knowing and a desire to close specific knowledge gaps (Litman, 2008). D‐type curiosity has been linked to question‐asking, targeted evidence seeking and effective experimental exploration in inquiry‐based learning (Jirout & Klahr, 2012; van Schijndel et al., 2018). These behaviours constitute proximal processes that support core aspects of scientific reasoning. I‐type curiosity, in contrast, is associated with broad engagement, positive affect and sustained exploration of content‐rich materials and is therefore thought to facilitate the gradual accumulation of conceptual knowledge (Hidi & Renninger, 2006; Renninger & Hidi, 2016).

Findings from cognitive neuroscience and educational psychology further inform about the possible mechanisms by which curiosity supports learning: Experimental work on state curiosity shows that periods of heightened curiosity enhance memory encoding (Gruber et al., 2014) and later retention (Fandakova & Gruber, 2021). Although these studies focus on state rather than trait curiosity, they reveal processes such as increased attention, reward‐related processing and deeper elaboration that can plausibly underlie more stable curiosity dispositions as well. Recent evidence links trait epistemic curiosity to sustained exploration and long‐term cognitive outcomes, such as cognitive reserve in adulthood (Wiegand et al., 2025). Together with developmental work showing relative stability of epistemic curiosity across childhood (Iwasaki et al., 2023; Piotrowski et al., 2014), this suggests that state and trait curiosity are related expressions of shared motivational‐cognitive mechanisms.

Despite these advances, important research gaps remain. First, there are still few longitudinal studies that examine how curiosity relates to the development of science competencies across the primary‐school years, a period marked by rapid changes in metacognition, self‐regulation and motivational dispositions (Eccles, 2005; Kidd & Hayden, 2015). Existing work has mainly focused on short‐term inquiry tasks or cross‐sectional associations between curiosity and performance (e.g., Jirout & Klahr, 2012; Lamnina & Chase, 2019, 2021; van Schijndel et al., 2018). Second, scientific reasoning and science knowledge are rarely considered as related but separate components of scientific literacy, even though they may follow partially independent developmental trajectories. Third, the differential contributions of I‐type and D‐type curiosity to these competencies remain largely unexplored in longitudinal designs, particularly when controlling for prior competence and cognitive ability.

The present study addresses these gaps by examining how overall epistemic curiosity, I‐type curiosity and D‐type curiosity measured at the beginning of primary school predict the development of scientific reasoning and science knowledge from Grade 1 to Grades 3 and 4. Using a longitudinal design with repeated assessments of scientific reasoning and science knowledge, and parent‐reported curiosity as a relatively stable disposition, we ask (1) how I‐type and D‐type curiosity relate to the growth of scientific reasoning and science knowledge over time, and (2) whether these curiosity facets explain variance beyond prior competence and non‐verbal intelligence. By differentiating between curiosity subtypes and between scientific reasoning and science knowledge, this study aims to clarify the motivational pathways through which curiosity supports children's science learning across development.

## MATERIALS AND METHODS

The present study was part of a larger longitudinal project investigating the development of scientific reasoning in early and middle childhood (Osterhaus & Koerber, 2023).

### Participants

Participants were 122 children (71 girls, 51 boys) with a mean age of 6 years 2 months (SD = 3.65 months) at the end of their final kindergarten year. Children were recruited in two cohorts, with Cohort 1 (C1) having their final kindergarten year in 2014 and Cohort 2 (C2) in 2015. Kindergartens were recruited through purposive regional sampling to ensure a heterogeneous yet comparable sample from urban (C1) and nearby rural areas (C2). Within each kindergarten, all children in their final preschool year and their parents were invited to participate. Data were collected at multiple time points, with the first wave taking place shortly before school entry (kindergarten) and yearly follow‐up assessments until Grade 4.

The two cohorts did not differ in their scientific reasoning, science knowledge nor in their curiosity (see Results). A language other than German was spoken at home by 14 children (11.6%). All procedures complied with the ethical standards prevailing at the time of data collection, including the principles of the Declaration of Helsinki and relevant national regulations. Ethical approval for this school‐based study was obtained from the responsible regional educational authority, which was the legally competent body for approving educational research at that time. Written parental consent and child assent were obtained. Participation was voluntary, and no monetary incentives were provided.

### Measures

Scientific reasoning and science knowledge were measured annually toward the end of each school year from kindergarten to Grade 4. This timing was chosen to capture developmental progress across major educational transitions and to ensure comparability across cohorts, as instructional experiences and content coverage were stable at these points in the academic year. (For a more comprehensive overview of the testing instruments and time see the Osterhaus & Koerber, 2023).

#### Scientific reasoning

**FIGURE 1:**
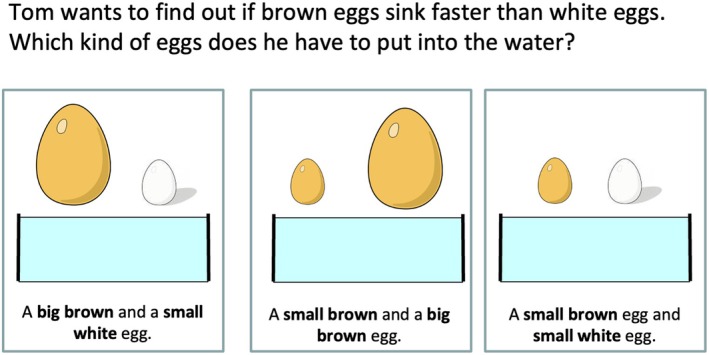
Sample item from the SKI assessing children's mastery of the control‐of‐variables strategy (experimentation). Reprinted from Koerber and Osterhaus (2019).

Scientific reasoning was assessed using the Science‐Kindergarten Inventory (SK‐I; Koerber & Osterhaus, 2019; see Figure 1) comprising 30 items, and five items from the Science‐Primary School Reasoning Inventory (SPR‐I; Osterhaus et al., 2020). The SK‐I was administered in an interview format up to Grade 2. From Grade 3 onward, 15 SK‐I items continued to be assessed via interviews, while the remaining 15 items were administered in a whole‐class testing format, and SPR‐I items were added. Ability estimates were obtained using weighted likelihood estimation (WLE; person‐separation reliability = .83; Osterhaus & Koerber, 2023).

#### Science knowledge

**FIGURE 2:**
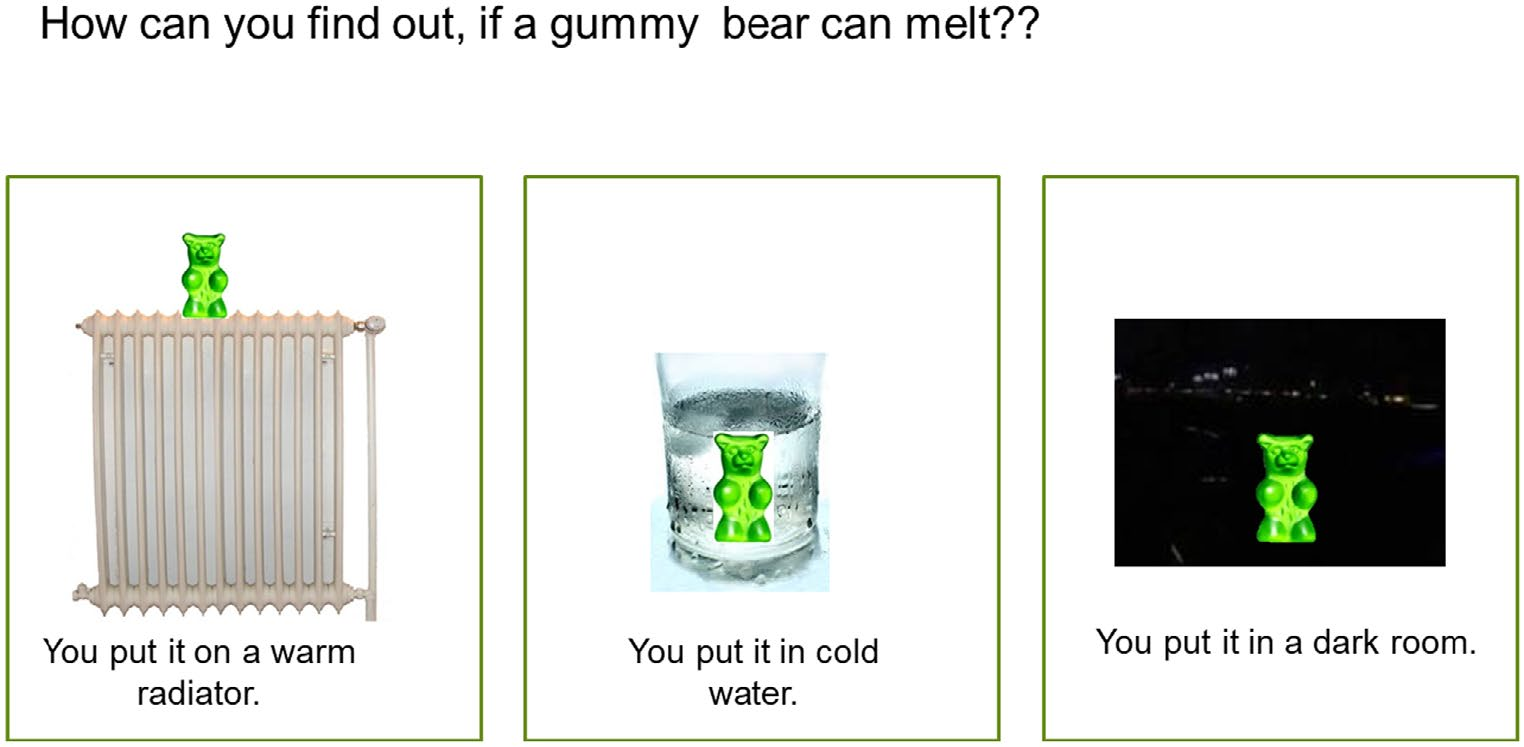
Sample item assessing children's knowledge on melting and evaporation (science knowledge).

Science knowledge was assessed with 25 items from two instruments: SNaKE (Carstensen et al., 2011; 21 items on melting and evaporation, slightly adapted for the present study; for an example see Figure 2) and Science‐P (Pollmeier et al., 2017; four items on density and displacement). At the end of kindergarten, all SNaKE items were administered, with four items retained through Grade 4. From Grade 2 onwards, four more advanced displacement items (Science‐P) were added. Items were Rasch‐scaled across five waves, showing acceptable model fit and moderate person‐separation reliability (0.60). Item difficulties aligned well with person abilities, with Science‐P items being the most challenging.

#### Epistemic curiosity

Parents completed a 10‐item questionnaire assessing epistemic curiosity (Litman, 2005; Piotrowski et al., 2014) on a five‐point Likert scale, supplemented by an additional item measuring their child's interest‐type science curiosity (‘I believe that my child is very curious about science topics’). The Litman‐scale differentiates between two dimensions of epistemic curiosity: interest‐type curiosity (I‐type; five items; e.g., ‘My child has fun learning about new topics or subjects’) and deprivation‐type curiosity (D‐type; five items; e.g., ‘My child is bothered when he or she does not understand something and tries hard to make sense of it’).

The interest‐type science curiosity item captures a domain‐related facet of I‐type curiosity and was included both in the overall curiosity composite scale (11 items) and in the I‐Type curiosity subscale, reflecting its conceptual alignment with the interest‐type dimension. Parents completed the questionnaire once when children were in Grade 1 (C2) or Grade 2 (C1). The two cohorts did not significantly differ in curiosity (see Table 1), supporting the comparability of assessments across cohorts. This single administration was chosen, as epistemic curiosity in childhood is considered a relatively stable dispositional construct (Jirout & Klahr, 2012; Piotrowski et al., 2014) and shows trait‐like consistency across early and middle childhood (Iwasaki et al., 2023). Details on the reliability of the scales are provided in the Results section and Table 1.

**TABLE 1:** Means and standard deviations for all measures assessed.

	*n*	*M*	*SD*	Cronbachs α	*t*‐test (cohort effects)
Curiosity in %					
**Curiosity all11** (11: I‐/D‐Type, Science curiosity)	**122**	**60.77**	**15.14**	.**816**	*t*(120) = −.469, *p* = .640, *d* = −0.09
**C1:** Curiosity **all11**	61	60.13	15.64	.812	
**C2**: Curiosity **all11**	61	61.42	14.72	.824	
**Curiosity all10** (10: I‐ /D‐Type, Litman Scale items)	**122**	**61.10**	**14.72**	.**791**	*t*(120) = −.657, *p* = .512, *d* = −0.12
**C1:** Curiosity **all10**	61	60.22	15.22	.789	
**C2**: Curiosity **all10**	61	61.98	14.27	.797	
**I‐Type Curiosity 5** (5 items)	**122**	**72.05**	**15.82**	.**603**	*t*(120) = −1.182, *p* = .240; *d* = −0.22
**C1:** I‐Type Curiosity **5**	61	70.35	16.43	.607	
**C2**: I‐Type Curiosity **5**	61	73.74	15.12	.591	
**Science_Curiosity** (1 item)	**121**	**57.85**	29.11		*t*(119) = .595, *p* = .553; *d* = 0.11
**C1:** Science_Curiosity 1	60	59.44	30.12		
**C2**: Science_Curiosity 1	61	56.28	28.25		
**I‐Type Curiosity 6** (I‐Type, + Science curiosity)	**122**	**69.64**	**16.09**	.**669**	*t*(120) = −.768, *p* = .444; *d* = −0.14
**C1:** I‐Type Curiosity **6**	61	68.52	16.68	.673	
**C2**: I‐Type Curiosity **6**	61	70.77	15.52	.663	
**D‐Type Curiosity** (5 items)	**122**	**49.92**	**17.40**	.**754**	*t*(120) = −.040, *p* = .968; *d* = −0.01
**C1:** D‐Type Curiosity **5**	61	49.85	17.81	.744	
**C2**: D‐Type Curiosity **5**	61	49.98	17.13	.781	
Scientific reasoning					
K1: Scientific Reasoning (2 PL)	120	−.494	.644		
G1: Scientific Reasoning (2 PL)	119	−.161	.646		
G2: Scientific Reasoning (2 PL)	112	.034	.897		
G3: Scientific Reasoning (2 PL)	116	.468	.615		
G4: Scientific Reasoning (2 PL)	112	.798	.591		
Science knowledge					
K1: Science Knowledge (WLE)	102	−.312	.838		
G1: Science Knowledge (WLE)	92	−547	.998		
G2: Science Knowledge (WLE)	101	1.580	1.179		
G3: Science Knowledge (WLE)	98	1.939	1.118		
G4: Science Knowledge (WLE)	83	1.985	1.025		

*Note*: Bold rows report descriptive statistics for the combined sample (C1 + C2). Rows below show the corresponding values for each cohort separately. T‐tests indicate that cohorts did not differ significantly.

#### Intelligence

Children's non‐verbal intelligence was assessed using the 10‐item ‘analogical reasoning’ subtest from the Vienna Development Test (WET Kastner‐Koller & Deimann, 2002) and the 15‐item ‘progressive matrices’ subtest from the Culture Fair Intelligence Test (CFT, Weiß, 2006). Both tasks involved figural reasoning and pattern recognition. Internal consistencies were acceptable, with Cronbach's α = .78 for the analogical reasoning subtest and α = .70 for the CFIT matrices.

All measurement instruments had been previously validated in large‐scale studies demonstrating sound construct and criterion validity (for scientific reasoning and science knowledge, see Koerber & Osterhaus, 2019; Osterhaus et al., 2020; for curiosity, see Piotrowski et al., 2014; for intelligence, see Kastner‐Koller & Deimann, 2002; Weiß, 2006). The present study replicated the expected intercorrelations among main study constructs (see Results and Table 2), supporting their validity within this sample.

**TABLE 2:** Correlations between curiosity and scientific reasoning (SR) as well as science knowledge (SK) for each grade.

	SR‐G1	SR‐G3	SR‐G4	SK‐G1	SK‐G3	SK‐G4
Cu_all	.343 (<.001)	.284 (.002)	.138 (.138)	.236 (.024)	.267 (.008)	.279 (.011)
Cu_I	.380 (<.001)	.281 (.002)	.179 (.060)	.254 (.015)	.342 (<.001)	.276 (.012)
Cu_D	.233 (.011)	.231 (.013)	.067 (.481)	.162 (.123)	.132 (.196)	.225 (.041)
Int. (K)	.184 (.050)	.136 (.154)	.128 (.189)	−.095 (.378)	−.031 (.765)	.127 (.260)

Abbreviations: Cu_all, Curiosity full scale of all 11 items; Cu_D, Curiosity Deprivation Type (Litman scale 5 items); Cu_I, Curiosity I‐Type (5 items from the Litman scale I‐Type scientific curiosity); Cu_Sci, Curiosity for Science (1 item); SK, science knowledge; SR, scientific reasoning; G, grade.

### Statistical analyses

All analyses were conducted using R and SPSS. Rasch‐scaled ability estimates (WLEs) based on 2PL models were taken from our previous validation studies (see Osterhaus & Koerber, 2023). We computed descriptive statistics, reliability estimates and correlations. Hierarchical regressions tested the predictive role of epistemic curiosity for scientific reasoning and science knowledge, controlling for prior competence and intelligence (*p* < .05, two‐tailed).

### Procedure

All individual interviews were conducted by trained researchers on separate days in quiet rooms at their respective kindergartens or schools (teachers were absent during the interviews). In fourth and third grade individual booklets and a simultaneous PowerPoint presentation were used during whole‐class assessment. Apart from the experimentator one or two test assistants were present to guarantee individual and quiet work of the children.

## RESULTS

### Core values in epistemic curiosity and science competencies

Table 1 presents descriptive statistics for all curiosity scales as well as the scores for the longitudinally measured scientific reasoning and science knowledge scales. Parents' estimation of children's epistemic curiosity yielded an average of 60.77% (SD = 15.14) for the full composite scale and 69.64% (SD = 16.09) and 49.92% (SD = 17.40) for the I‐type curiosity and for the D‐type curiosity, respectively.

There were no differences in epistemic curiosity between both cohorts (see Table 1), nor between gender in any of the curiosity components (all effects were small |*d*| ≤ .22) [*t*(120) = .547, *p* = .586; *t*(120) = −.241, *p* = .810; *t*(120) = .928, *p* = .355; *t*(119) = .982, *p* = .374 for the full composite scale, the I‐Type Curiosity, D‐Type Curiosity and the I‐Type Science Curiosity single item, respectively]. As no cohort nor gender differences were found for any of the subscales, reliability analyses (Cronbach's alpha statistics) are based on the full sample.

All scales showed acceptable to good internal consistency (α ≥ .75), except the five‐item I‐Type curiosity scale (α = .603), which improved with inclusion of the I‐Type science‐related item (six items; α = .669). Accordingly, the six‐item I‐Type scale (enhanced reliability and conceptual fit) and the 11‐item composite were used in all subsequent analyses, along with the D‐Type scale.

### Correlations between epistemic curiosity and science competencies

Table 2 presents the correlations between different facets of epistemic curiosity, intelligence and scientific reasoning and science knowledge at three key stages of primary school: Grade 1 (entry), Grade 3 and Grade 4 (end of primary school).

From a developmental perspective, the strongest and broadest associations between epistemic curiosity and scientific reasoning emerged in Grade 1, particularly for the composite curiosity scales (*r* = .343, *p* < .001). These associations remained significant but were weaker in Grade 3 and were no longer present in Grade 4, suggesting that the influence of general epistemic curiosity on scientific reasoning may be most pronounced in early primary school, with all subtypes, including I‐type and D‐type, showing similar correlations; though contrary to expectations, D‐type curiosity did not stand out as particularly strong.

In contrast, science knowledge showed a more stable pattern of associations across grades, especially with I‐type curiosity. The I‐type scale correlated significantly with science knowledge at each timepoint (*r*s between .254 and .342), indicating that children who show sustained, interest‐driven curiosity tend to accumulate more factual science knowledge over time.

Interestingly, D‐Type curiosity (focused on resolving knowledge gaps) displayed a less consistent pattern. While it was modestly associated with scientific reasoning in Grades 1 and 3, its link to scientific reasoning disappeared by Grade 4, whereas a small but significant correlation with science knowledge emerged only at this later stage (*r* = .225, *p* = .041). Notably, in Grade 1, the correlation with scientific reasoning was significantly stronger than with science knowledge (*z* = −3.59, *p* < .001), whereas in Grade 4, this pattern reversed, with a significantly stronger association with science knowledge than with scientific reasoning (*z* = −2.17, *p* = .030). This may suggest a possible developmental shift: children may increasingly apply this form of curiosity toward acquiring factual knowledge, while its relevance for reasoning‐based tasks appears to decline.

It should be noted that intelligence showed only weak or non‐significant correlations with both scientific reasoning and science knowledge across all grades (*r*s < .20, all *p*s > .05), except for a small association with Grade 1 scientific reasoning (*r* = .18, *p* = .05, see Table 2).

Overall, the findings highlight a differentiation between scientific reasoning and science knowledge in how they relate to different curiosity facets over time. Scientific reasoning appears to benefit particularly from broad, general epistemic curiosity in early school years, whereas science knowledge is linked to curiosity throughout primary school, with an especially strong link to I‐type curiosity.

### Predicting third and fourth grade science competencies from children's epistemic curiosity

To examine the predictive role of epistemic curiosity beyond prior knowledge and intelligence, a series of hierarchical linear regressions were conducted for scientific reasoning and science knowledge across primary school. Intelligence and science knowledge assessed in kindergarten were entered in Step 1, followed by curiosity measures in Step 2. Table 3 gives an overview, summarizing the standardized regression coefficients (*β*), incremental explained variances (Δ*R* ^2^) and significance levels (*p*) for each curiosity scale across grades. Tables 4 and 5 give detailed information concerning two regressions analyses of curiosity on scientific reasoning (Table 4) and on science knowledge (Table 5).

**TABLE 3:** Overview of different regressions from three different scales of curiosity to SR and SK for each grade, with kindergarten intelligence and prior reasoning/prior knowledge considered in a first model = > line one beta (p) and delta *R* ^2^.

		SR‐G1	SR‐G3	SR‐G4	SK‐G1	SK‐G3	SK‐G4
Cu_all	*β*	.197 (.028)	.177 (.041)	.031 (.725)	.191 (.093)	.134 (.241)	.219 (.068)
	Δ*R* ^2^	.036	.029	.001	.025	.017	.047
Cu_I	*β*	.205 (.015)	.146 (.093)	−.034 (.708)	.230 (.045)	.248 (.027)	.229^#^(.056)
	Δ*R* ^2^	.037	.020	.001	.049	.058	.051
Cu_D	*β*	.147 (.066)	.167 (.048)	−.023 (.793)	.108 (.337)	−.021 (.852)	.106 (.184)
	Δ*R* ^2^	.021	.027	.001	.011	.000	−.025

*Note*: The symbol “#” indicates marginal significance (*p* < .10).

Abbreviations: Cu_all, Curiosity full scale of all 11 items; Cu_D, Curiosity Deprivation Type (Litman scale 5 items); Cu_I, Curiosity I‐Type (5 items from the Litman scale I‐Type scientific curiosity); Cu_Sci, Curiosity for Science (1 item); SK, science knowledge; SR, scientific reasoning.

**TABLE 4:** Results of a hierarchical regression analysis: Predicting scientific reasoning in first, third and fourth grades.

	*β*	*t*	*p*	Change in *F*	Corrected *R* ^2^
DV = Scientific reasoning in 1st Grade					
Model 1				*F*(2, 110) = 24.098	.292
Scientific reasoning (K)	.527	6.597	<.001		
Intelligence (K)	.124	1.555	.123		
Model 2				*F*(1, 109) = 5.961	.323
Scientific reasoning (K)	.475	5.861	<.001		
Intelligence (K)	.130	1.668	.098		
Curiosity (gen.)	.197	2.442	.016		
DV = Scientific reasoning in 3rd Grade					
Model 1				*F*(2, 107) = 17.448	.232
Scientific reasoning (K)	.482	5.727	<.001		
Intelligence (K)	.089	1.063	.290		
Model 2				*F*(1, 106) = 4.292	.255
Scientific reasoning (K)	.437	5.105	<.001		
Intelligence (K)	.100	1.202	.232		
Curiosity (gen.)	.177	2.072	.041		
DV = Scientific reasoning in 4th Grade					
Model 1				*F*(2, 103) = 17.130	.235
Scientific reasoning (K)	.485	5.669	*<.001*		
Intelligence (K)	.098	1.144	.*255*		
Model 2				*F*(1, 102) = .124	.228
Scientific reasoning (K)	.492	5.551	*<.001*		
Intelligence (K)	.096	1.114	.268		
Curiosity (gen.)	−.031	−.352	.725		

**TABLE 5:** Results of a hierarchical regression analysis: predicting science knowledge in first, third and fourth grades.

	*β* (standardized)	*t*	*p*	Change in *F*	Corrected *R* ^2^
DV = Science knowledge in 1st Grade					
Model 1				*F*(2, 76) = 2.796	.044
Science knowledge (K)	.254	2.251	.027		
Intelligence (K)	−.127	−1.129	.262		
Model 2c				*F*(1, 75) = 4.174	.082
Science knowledge (K)	.190	1.656	.102		
Intelligence (K)	−.117	−1.060	.293		
Curiosity (I‐type6)	.230	2.043	.045		
DV = Science knowledge in 3rd Grade					
Model 1				*F*(2, 78) = 2.751	.042
Science knowledge (K)	.229	2.069	.042		
Intelligence (K)	−.153	−1.383	.170		
Model 2				*F*(1, 77) = 5.062	.089
Science knowledge (K)	.170	1.530	.130		
Intelligence (K)	−.121	−1.115	.268		
Curiosity (I‐type6)	.248	2.250	.027		
DV = Science knowledge in 4th Grade					
Model 1				*F*(2, 68) = 1.443	.013
Science knowledge (K)	.176	1.449	.152		
Intelligence (K)	.069	.568	.572		
Model 2				*F*(1, 67) = 3.788	.051
Science knowledge (K)	.143	1.193	.237		
Intelligence (K)	.087	.728	.469		
Curiosity (I‐type6)	.229	1.946	.056		

#### Scientific reasoning

In Grades 1 and 3, kindergarten scientific reasoning and intelligence accounted for a substantial portion of variance (Adj. *R* ^2^ = .29 and .23, respectively, both *p* < .001). Adding general epistemic curiosity (11‐item scale) significantly improved the model (Δ*F*(1, 109) = 5.961, *p* = .016 and Δ*F*(1, 106) = 4.292, *p* = .041 for Grades 1 and 3, respectively) with curiosity contributing uniquely to scientific reasoning (*β* = .197, *p* = .016 and *β* = .177, *p* = .041 for Grades 1 and 3, respectively). The overall model explained 32% and 26% of the variance in Grades 1 and 3, respectively. By Grade 4, general epistemic curiosity showed no predictive effects on scientific reasoning anymore. This suggests a developmental decline in the predictive power of curiosity for scientific reasoning.

#### Science knowledge

In Grade 1, I‐Type curiosity explained a significant proportion of additional variance in science knowledge after accounting for kindergarten (prior science knowledge and intelligence) predictors (Δ*R* ^2^ = .049, *ΔF*(1, 75) = 4.17, *p* = .045). Curiosity emerged as a significant unique predictor (*β* = .23, *p* = .045), while prior predictors became non‐significant in the final model. The overall model explained 8.2% of the variance (Adj. *R* ^2^ = .082). Similarly, in Grade 3, curiosity again accounted for significant incremental variance (Δ*R* ^2^ = .058, *ΔF*(1, 77) = 5.06, *p* = .027), increasing explained variance from 4.2% to 8.9% (Adj. *R* ^2^ = .089). Curiosity remained a significant predictor (*β* = .25, *p* = .027). By Grade 4, I‐Type curiosity still showed a marginal predictive effect (*β* = .23, *p* = .056; Δ*F*(1, 67) = 3.79, *p* = .056). The overall model explained 5.1% of the variance in science knowledge (Adj. *R* ^2^ = .051), suggesting a sustained though slightly reduced influence of early I‐Type curiosity on later SK.

## DISCUSSION

This longitudinal study examined how I‐ vs. D‐type epistemic curiosity predicts the development of scientific reasoning and science knowledge across primary school years. Our findings indicate that these curiosity types support different components of science learning through distinct mechanisms and developmental pathways. Scientific reasoning was predicted by overall curiosity rather than D‐type alone in Grades 1 and 3, whereas science knowledge was robustly predicted by I‐type curiosity across Grades 1, 3 and 4.

### I‐type and D‐type curiosity

Previous literature often emphasizes D‐type curiosity as the primary developmental motor of scientific reasoning. D‐type curiosity, which is typically triggered by the aversive feeling of not knowing, has been associated with targeted question‐asking, hypothesis testing and evidence‐seeking behaviours (Jirout & Klahr, 2012; Loewenstein, 1994). Our findings challenge and refine this view by showing that while D‐type curiosity does contribute to early scientific reasoning, it is not sufficient on its own. Rather, scientific reasoning benefits most from the combination of I‐type and D‐type curiosity, particularly in the early grades, when children's metacognitive strategies and cognitive control are still developing. I‐type curiosity, motivated by the intrinsic enjoyment of learning, sustains engagement, promotes broad exploration and provides the cognitive scaffolding necessary for effective problem‐solving. In this way, early scientific reasoning emerges from the interplay of both curiosity types, bridging intuitive exploration and more structured reasoning (Gopnik & Schulz, 2004).

In contrast, science knowledge was associated with I‐type curiosity. By promoting broad engagement and the intrinsic enjoyment of learning, I‐type curiosity may support sustained exploration and cumulative knowledge growth (Hidi & Renninger, 2006; Renninger & Hidi, 2016; Tang & Salmela‐Aro, 2021). This interpretation aligns with evidence from cognitive neuroscience showing that periods of heightened curiosity enhance attention, memory encoding and later retention (Fandakova & Gruber, 2021; Gruber et al., 2014), suggesting that intrinsically motivated exploration lays the foundation for long‐term conceptual development. Our results also echo findings that trait epistemic curiosity predicts cognitive reserve (Wiegand et al., 2025), emphasizing the developmental significance of curiosity beyond momentary task performance.

Taken together, our study suggests that curiosity subtypes uniquely contribute to science competence. D‐type curiosity, while previously regarded as central to scientific reasoning, requires the support of I‐type curiosity to translate into effective reasoning behaviours in young children. I‐type curiosity, in contrast, exerts a robust and consistent influence on science knowledge, observable across multiple grades.

### Developmental patterns

Our findings demonstrate that scientific reasoning and science knowledge follow partially independent developmental pathways. In early primary school, scientific reasoning is driven by the co‐occurrence of curiosity types, whereas science knowledge is consistently predicted by I‐type curiosity alone.

With respect to scientific reasoning, this likely reflects that younger children's inquiry remains closely tied to resolving immediate uncertainty and exploring novel phenomena. At this stage, scientific reasoning is not yet formalized but often embedded in spontaneous exploration and early question‐asking, a pattern also supported by evidence that children naturally explore more than adults (Liquin & Gopnik, 2022). The co‐occurrence of I‐ and D‐type curiosity may scaffold the transition from intuitive exploration to more structured reasoning. Specifically, I‐type curiosity may amplify the effects of D‐type curiosity by promoting sustained engagement, open‐ended exploration and the cognitive scaffolding necessary for effective problem‐solving and hypothesis testing (Loewenstein, 1994). Prior work further suggests that such situated, exploratory curiosity provides a foundation for later structured reasoning (Peterson & Cohen, 2019, here with respect to mathematics). Our findings extend this perspective by showing how I‐type and D‐type curiosity unfold differently across science competencies.

In contrast, science knowledge is stably supported by I‐type curiosity from Grades 1 to 4, including more advanced items. Interest‐driven engagement promotes deeper conceptual processing and long‐term knowledge integration. As metacognitive abilities (e.g., uncertainty monitoring; Goupil & Proust, 2023) mature, I‐type curiosity increasingly enables children to seek out complex information even without immediate gaps.

Together, these divergent pathways show that curiosity exerts domain‐specific and developmentally shifting influences on science learning. D‐type curiosity facilitates short‐term inferential behaviour under uncertainty, whereas I‐type curiosity supports long‐term conceptual acquisition, particularly in domains that demand abstract reasoning and the integration of complex information (e.g., Fandakova & Gruber, 2021; Hidi & Renninger, 2006; Lamnina & Chase, 2019). Recognizing this distinction underscores the need to treat scientific reasoning and science knowledge as separate yet interrelated competencies, each shaped by distinct curiosity profiles across development.

Finally, these findings align with theoretical models distinguishing early curiosity as an affectively charged response to novelty from later curiosity as a sustained cognitive orientation toward deep learning (Kidd & Hayden, 2015). Our results reinforce that epistemic curiosity is not monolithic but evolves across childhood, differently shaping distinct components of science learning.

### The role of intelligence

Although intelligence is often regarded as a key determinant of academic achievement, it contributed only marginally to either outcome once prior knowledge and curiosity were accounted for. This aligns with findings that the predictive strength of intelligence diminishes over time as motivational and self‐regulatory factors become more influential (Steinmayr et al., 2019). Moreover, although intelligence typically explains large portions of variance in academic outcomes (Kriegbaum et al., 2018), its predictive contribution to domain‐specific learning, such as scientific reasoning or science knowledge acquisition, appears to be overshadowed when curiosity and prior conceptual understanding are considered (van Schijndel et al., 2018).

### Limitations

Notwithstanding the longitudinal design and theoretical differentiation, two limitations should be noted. First, epistemic curiosity was assessed only once at school entry using parental reports, which constrains conclusions about developmental change, state–trait differentiation and its distinction from related constructs such as interest. Future studies should therefore employ repeated, multi‐method assessments to capture how different facets of curiosity unfold across the school years. Second, effect sizes were modest but consistent across grades and outcomes, underscoring the developmental relevance of curiosity beyond intelligence and prior knowledge.

### Conclusions and implications

In terms of contribution to knowledge, this study demonstrates that epistemic curiosity is a functionally differentiated motivational resource, operating through distinct faces, I‐type and D‐type curiosity (Litman, 2008), whose relevance varies across developmental phases and science competencies. Scientific reasoning in early primary school was supported by the joint contribution of both curiosity faces, indicating that information‐gap driven inquiry (Loewenstein, 1994) yields reasoning gains only when embedded in sustained interest‐based engagement. In contrast, science knowledge was selectively and robustly associated with I‐type curiosity across Grades 1–4, consistent with developmental models of interest‐driven learning (Hidi & Renninger, 2006) and accounts of curiosity as a stable motivational orientation (Kidd & Hayden, 2015). Together, these findings provide evidence for a developmental reweighting account in which curiosity faces differentially support scientific reasoning and science knowledge across childhood.

In terms of contribution to practise, the results indicate that effective support of curiosity in science learning requires aligning curiosity faces with developmental phases and competency demands, rather than relying on generic curiosity stimulation.

Taken together, these findings underscore that epistemic curiosity is not a unitary trait but a developmentally dynamic motivational resource that differentially shapes distinct components of science learning across childhood.

## AUTHOR CONTRIBUTIONS

**Susanne Koerber:** Writing – original draft; formal analysis; conceptualization; supervision; funding acquisition; investigation; methodology; visualization; writing – review and editing; project administration. **Christopher Osterhaus:** Writing – review and editing; data curation; investigation; methodology; conceptualization; formal analysis; validation; project administration.

## CONFLICT OF INTEREST STATEMENT

The authors declare no conflicts of interest.

## Data Availability

The data that support the findings of this study are available from the corresponding author upon reasonable request.

## References

[bjdp70041-bib-0001] Bjerknes, A. L. , Wilhelmsen, T. , & Foyn‐Bruun, E. (2023). A systematic review of curiosity and wonder in natural science and early childhood education research. Journal of Research in Childhood Education, 38(1), 50–65. 10.1080/02568543.2023.2192249

[bjdp70041-bib-0002] Carstensen, C. H. , Lankes, E.‐M. , & Steffensky, M. (2011). Ein Modell zur Erfassung naturwissenschaftlicher Kompetenz im Kindergarten. Zeitschrift für Erziehungswissenschaft, 14(4), 651–669. 10.1007/s11618-011-0204-2

[bjdp70041-bib-0003] Eccles, J. S. (2005). Subjective task value and the Eccles et al. model of achievement‐related choices. In A. J. Elliot & C. S. Dweck (Eds.), Handbook of competence and motivation (pp. 105–121). Guilford Publications.

[bjdp70041-bib-0004] Fandakova, Y. , & Gruber, M. J. (2021). States of curiosity and interest enhance memory differently in adolescents and in children. Developmental Science, 24(1), e13005. 10.1111/desc.13005 32524703 PMC7618219

[bjdp70041-bib-0005] Fischer, F. , Chinn, C. A. , Engelmann, K. , & Osborne, J. (2018). Scientific reasoning and argumentation: The roles of domain‐specific and domain‐general knowledge. Routledge.

[bjdp70041-bib-0006] Gopnik, A. , & Schulz, L. (2004). Mechanisms of theory formation in young children. Trends in Cognitive Sciences, 8(8), 371–377. 10.1016/j.tics.2004.06.005 15335464

[bjdp70041-bib-0007] Goupil, L. , & Proust, J. (2023). Curiosity as a metacognitive feeling. Cognition, 231, 105325. 10.1016/j.cognition.2022.105325 36434942

[bjdp70041-bib-0008] Gruber, M. J. , Gelman, B. D. , & Ranganath, C. (2014). States of curiosity modulate hippocampus‐dependent learning via the dopaminergic circuit. Neuron, 84(2), 486–496. 10.1016/j.neuron.2014.08.060 25284006 PMC4252494

[bjdp70041-bib-0009] Hartung, F. M. , Thieme, P. , Wild‐Wall, N. , & Hell, B. (2022). Being snoopy and smart. Journal of Individual Differences, 43(4), 194–201. 10.1027/1614-0001/a000372

[bjdp70041-bib-0010] Hidi, S. , & Renninger, K. A. (2006). The four‐phase model of interest development. Educational Psychologist, 41(2), 111–127. 10.1207/s15326985ep4102_4

[bjdp70041-bib-0011] Iwasaki, S. , Moriguchi, Y. , & Sekiyama, K. (2023). Parental responsiveness and children's trait epistemic curiosity. Frontiers in Psychology, 13, 1075489. 10.3389/fpsyg.2022.1075489 36778159 PMC9910790

[bjdp70041-bib-0012] Jirout, J. J. , Evans, N. S. , & Son, L. K. (2024). Curiosity in children across ages and contexts. Nature Reviews Psychology, 3(9), 622–635. 10.1038/s44159-024-00346-5

[bjdp70041-bib-0013] Jirout, J. J. , & Klahr, D. (2012). Children's scientific curiosity: In search of an operational definition of an elusive concept. Developmental Review, 32(2), 125–160. 10.1016/j.dr.2012.04.002

[bjdp70041-bib-0014] Kastner‐Koller, U. , & Deimann, P. (2002). Wiener Entwicklungstest: Ein Verfahren zur Erfassung des allgemeinen Entwicklungsstandes bei Kindern von 3 bis 6 Jahren [The Vienna Development Test: An instrument to measure the general development of children aged 3 to 6]. Hogrefe.

[bjdp70041-bib-0015] Kidd, C. , & Hayden, B. Y. (2015). The psychology and neuroscience of curiosity. Neuron, 88(3), 449–460. 10.1016/j.neuron.2015.09.010 26539887 PMC4635443

[bjdp70041-bib-0016] Koerber, S. , & Osterhaus, C. (2019). Individual differences in early scientific thinking: Assessment, cognitive influences, and their relevance for science learning. Journal of Cognition and Development, 20(4), 510–533. 10.1080/15248372.2019.1620232

[bjdp70041-bib-0017] Kriegbaum, K. , Becker, N. , & Spinath, B. (2018). The relative importance of intelligence and motivation as predictors of school achievement: A meta‐analysis. Educational Research Review, 25, 120–148. 10.1016/j.edurev.2018.10.001

[bjdp70041-bib-0018] Kuhn, D. (2011). What is scientific thinking and how does it develop? In U. Goswami (Ed.), Handbook of childhood cognitive development (2nd ed., pp. 472–523). Blackwell.

[bjdp70041-bib-0019] Lamnina, M. , & Chase, C. C. (2019). Developing a thirst for knowledge: How uncertainty in the classroom influences curiosity, affect, learning, and transfer. Contemporary Educational Psychology, 59, 101785. 10.1016/j.cedpsych.2019.101785

[bjdp70041-bib-0020] Lamnina, M. , & Chase, C. C. (2021). Uncertain instruction: Effects on curiosity, learning, and transfer. Instructional Science, 49(5), 661–685. 10.1007/s11251-021-09557-2

[bjdp70041-bib-0021] Liquin, E. G. , & Gopnik, A. (2022). Children are more exploratory and learn more than adults in an approach‐avoid task. Cognition, 218, 104940. 10.1016/j.cognition.2021.104940 34715584

[bjdp70041-bib-0022] Litman, J. A. (2005). Curiosity and the pleasures of learning: Wanting and liking new information. Cognition and Emotion, 19(6), 793–814. 10.1080/02699930541000101

[bjdp70041-bib-0023] Litman, J. A. (2008). Interest and deprivation factors of epistemic curiosity. Personality and Individual Differences, 44(7), 1585–1595. 10.1016/j.paid.2008.01.014

[bjdp70041-bib-0024] Loewenstein, G. (1994). The psychology of curiosity: A review and reinterpretation. Psychological Bulletin, 116(1), 75–98. 10.1037/0033-2909.116.1.75

[bjdp70041-bib-0025] Osterhaus, C. , & Koerber, S. (2023). The complex associations between scientific reasoning and advanced theory of mind. Child Development, 94(1), e18–e42. 10.1111/cdev.13860 36321437

[bjdp70041-bib-0026] Osterhaus, C. , & Koerber, S. (2025). Domain‐general scientific reasoning abilities in kindergarten independently predict elementary‐school children's mathematics ability. British Journal of Developmental Psychology, 44(1), 134–145. 10.1111/bjdp.70013 40832926 PMC12884364

[bjdp70041-bib-0027] Osterhaus, C. , Koerber, S. , & Sodian, B. (2020). The science‐P reasoning inventory (SPR‐I): Measuring emerging scientific‐reasoning skills in primary school. International Journal of Science Education, 42(7), 1087–1107. 10.1080/09500693.2020.1748251

[bjdp70041-bib-0028] Peterson, E. G. , & Cohen, J. (2019). A case for domain‐specific curiosity in mathematics. Educational Psychology Review, 31, 807–832. 10.1007/s10648-019-09501-4

[bjdp70041-bib-0029] Piekny, J. , & Maehler, C. (2013). Scientific reasoning in early and middle childhood: The development of domain‐general evidence evaluation, experimentation, and hypothesis generation skills. British Journal of Developmental Psychology, 31(2), 153–179. 10.1111/j.2044-835X.2012.02082.x 23659889

[bjdp70041-bib-0030] Pintrich, P. R. (2000). The role of goal orientation in self‐regulated learning. In M. Boekaerts , P. R. Pintrich , & M. Zeidner (Eds.), Handbook of self‐regulation (pp. 451–502). Academic Press.

[bjdp70041-bib-0031] Piotrowski, J. T. , Litman, J. A. , & Valkenburg, P. (2014). Measuring epistemic curiosity in young children. Infant and Child Development, 23(5), 542–553. 10.1002/icd.1847

[bjdp70041-bib-0032] Pollmeier, J. , Tröbst, S. , Hardy, I. , Möller, K. , Kleickmann, T. , Jurecka, A. , & Schwippert, K. (2017). Science‐P I: Modeling conceptual understanding in primary school. In D. Leutner , J. Fleischer , J. Grünkorn , & E. Klieme (Eds.), Competence assessment in education: Research, models and instruments (pp. 9–17). Springer. 10.1007/978-3-319-50030-0

[bjdp70041-bib-0033] Renninger, K. A. , & Hidi, S. E. (2016). The power of interest for motivation and engagement. Routledge.

[bjdp70041-bib-0034] Sodian, B. (2018). The development of scientific thinking in preschool and elementary school age. A conceptual model. In F. Fischer , C. A. Chinn , K. Engelmann , & J. Osborne (Eds.), Scientific reasoning and argumentation. The roles of domain‐specific and domain‐general knowledge (pp. 227–250). Routledge.

[bjdp70041-bib-0035] Steinmayr, R. , Weidinger, A. F. , Schwinger, M. , & Spinath, B. (2019). The importance of students' motivation for their academic achievement – Replicating and extending previous findings. Frontiers in Psychology, 10, 1730. 10.3389/fpsyg.2019.01730 31417459 PMC6685139

[bjdp70041-bib-0036] Tang, X. , & Salmela‐Aro, K. (2021). The prospective role of epistemic curiosity in national standardized test performance. Learning and Individual Differences, 88, 102008. 10.1016/j.lindif.2021.102008

[bjdp70041-bib-0037] van der Graaf, J. , Segers, E. , & Verhoeven, L. (2018). Individual differences in the development of scientific thinking in kindergarten. Learning and Instruction, 56, 1–9. 10.1016/j.learninstruc.2018.03.005

[bjdp70041-bib-0038] van Schijndel, T. J. , Jansen, B. , & Raijmakers, M. (2018). Do individual differences in children's curiosity relate to their inquiry‐based learning? International Journal of Science Education, 40, 1–20. 10.1080/09500693.2018.1460772

[bjdp70041-bib-0039] van Schijndel, T. J. , & Jansen, B. R. (2025). Integrating lines of research on children's curiosity‐driven learning. Journal of Experimental Child Psychology, 252, 106168. 10.1016/j.jecp.2024.106168 39799780

[bjdp70041-bib-0040] van Vo, D. , & Csapó, B. (2023). Exploring inductive reasoning, scientific reasoning and science motivation, and their role in predicting STEM achievement across grade levels. International Journal of Science and Mathematics Education, 21(8), 2375–2398. 10.1007/s10763-022-10349-4

[bjdp70041-bib-0041] Veenman, M. V. J. (2013). Learning to self‐monitor and self‐regulate. In D. H. Schunk & B. J. Zimmerman (Eds.), Handbook of self‐regulation of learning and performance (2nd ed., pp. 83–101). Routledge.

[bjdp70041-bib-0042] Weiß, R. H. (2006). Grundintelligenztest Skala 2 (CFT 20‐R) mit Wortschatztest (WS) und Zahlenfolgentest (ZF)—Revision 2006 [basic intelligence scale 2 (CFT 20‐R) with vocabulary and digit span test — Revision 2006]. Hogrefe.

[bjdp70041-bib-0043] Wiegand, I. , Donkers, I. , Balart‐Sanchez, S. , Pope, M. , Pérez‐Ayora, G. , & Oosterman, J. M. (2025). The relationship between trait curiosity and cognitive reserve in younger and older adults. Scientific Reports, 15, 24707. 10.1038/s41598-025-10101-2 40634507 PMC12241511

[bjdp70041-bib-0044] Zimmerman, C. (2007). The development of scientific thinking skills in elementary and middle school. Developmental Review, 27(2), 172–223. 10.1016/j.dr.2006.12.001

